# Multilayered regulation of proteome stoichiometry

**DOI:** 10.1007/s00294-021-01205-z

**Published:** 2021-08-12

**Authors:** Koji Ishikawa

**Affiliations:** Center for Molecular Biology, ZMBH-DKFZ Alliance, Heidelberg University, Im Neuenheimer Feld 282, 69120 Heidelberg, Germany

**Keywords:** Yeast, Multiprotein complex, Stoichiometry, Protein degradation, Dosage compensation

## Abstract

Cellular systems depend on multiprotein complexes whose functionalities require defined stoichiometries of subunit proteins. Proper stoichiometry is achieved by controlling the amount of protein synthesis and degradation even in the presence of genetic perturbations caused by changes in gene dosage. As a consequence of increased gene copy number, excess subunits unassembled into the complex are synthesized and rapidly degraded by the ubiquitin–proteasome system. This mechanism, called protein-level dosage compensation, is widely observed not only under such perturbed conditions but also in unperturbed physiological cells. Recent studies have shown that recognition of unassembled subunits and their selective degradation are intricately regulated. This review summarizes the nature, strategies, and increasing complexity of protein-level dosage compensation and discusses possible mechanisms for controlling proteome stoichiometry in multiple layers of biological processes.

## Introduction

Cellular systems are exposed to a wide variety of environmental changes leading to fluctuations in gene expression and even cell fate (Elowitz et al. 2002; Raj et al. 2010; Raj and Van Oudenaarden 2008; Raser and O'Shea 2004, 2005). Robust control of intracellular concentrations of gene products for the proper rate of biological processes is a prerequisite for cellular systems. In this context, mechanisms buffering intrinsic and extrinsic perturbations in gene expression are the foundation for maintaining cellular homeostasis and ensure cell survival in response to various environments (Kitano 2007; Masel and Siegal 2009; Stelling et al. 2004). Indeed, recent studies have revealed that in *Saccharomyces cerevisiae*, transient gene dosage imbalance under physiological conditions, especially during DNA replication (early- and late-replicating genes) and meiosis, can be buffered by adjusting the rates of messenger RNA (mRNA) synthesis and protein degradation, respectively (Bar-Ziv et al. 2016; Eisenberg et al. 2018; Voichek et al. 2016). Furthermore, considering that an increase in the copy number of only a subset of the genome leads to a negative impact on cell growth in *S*. *cerevisiae* (Makanae et al. 2013; Sopko et al. 2006), genetic perturbations to a broad range of biological processes must be buffered by various mechanisms.

Genetic perturbations caused by increased gene dosage seem to be mainly buffered post-translationally in yeast and human cells (Dephoure et al. 2014; Stingele et al. 2012). Intracellular protein concentration is maintained at an appropriate level by the balance between protein synthesis and degradation. Genome-wide measurements of mRNA translation by ribosome profiling, which is the deep sequencing of ribosome-protected mRNA fragments (ribosome footprints), revealed that compared to mRNA abundance, ribosome footprint density is better correlated with protein abundance determined by mass spectrometry (Ingolia et al. 2009). Whereas translation efficiency, as measured by ribosome profiling, does not decrease upon an increase in gene dosage as described below, degradation of the resulting excess proteins is accelerated to adjust their concentration. These results indicate that the effects of changes in gene dosage are partially buffered by protein degradation, which is referred to as protein-level dosage compensation. Of note, although dosage compensation at the mRNA level under genetic perturbations is controversial, it has been argued that a feedback control of the mRNA level could occur to reduce protein synthesis (Veitia and Potier 2015).

Protein-level dosage compensation plays a crucial role in fine-tuning stoichiometry of multiprotein complex subunits that compose a large fraction of the proteome (Dephoure et al. 2014; Eisenberg et al. 2018; Ishikawa et al. 2017; Shemorry et al. 2013; Sung et al. 2016), thereby complementing the precisely controlled synthesis of those proteins and maintaining proteome stoichiometry (Ingolia et al. 2018). Although we know that protein degradation mediates the compensation, how unassembled subunits are selectively degraded remains largely an open question. This review presents a current overview of the stoichiometry control system, with the main focus on the nature, functions, and mechanisms of protein-level dosage compensation.

## Nature and functions of protein-level dosage compensation

**Fig. 1:**
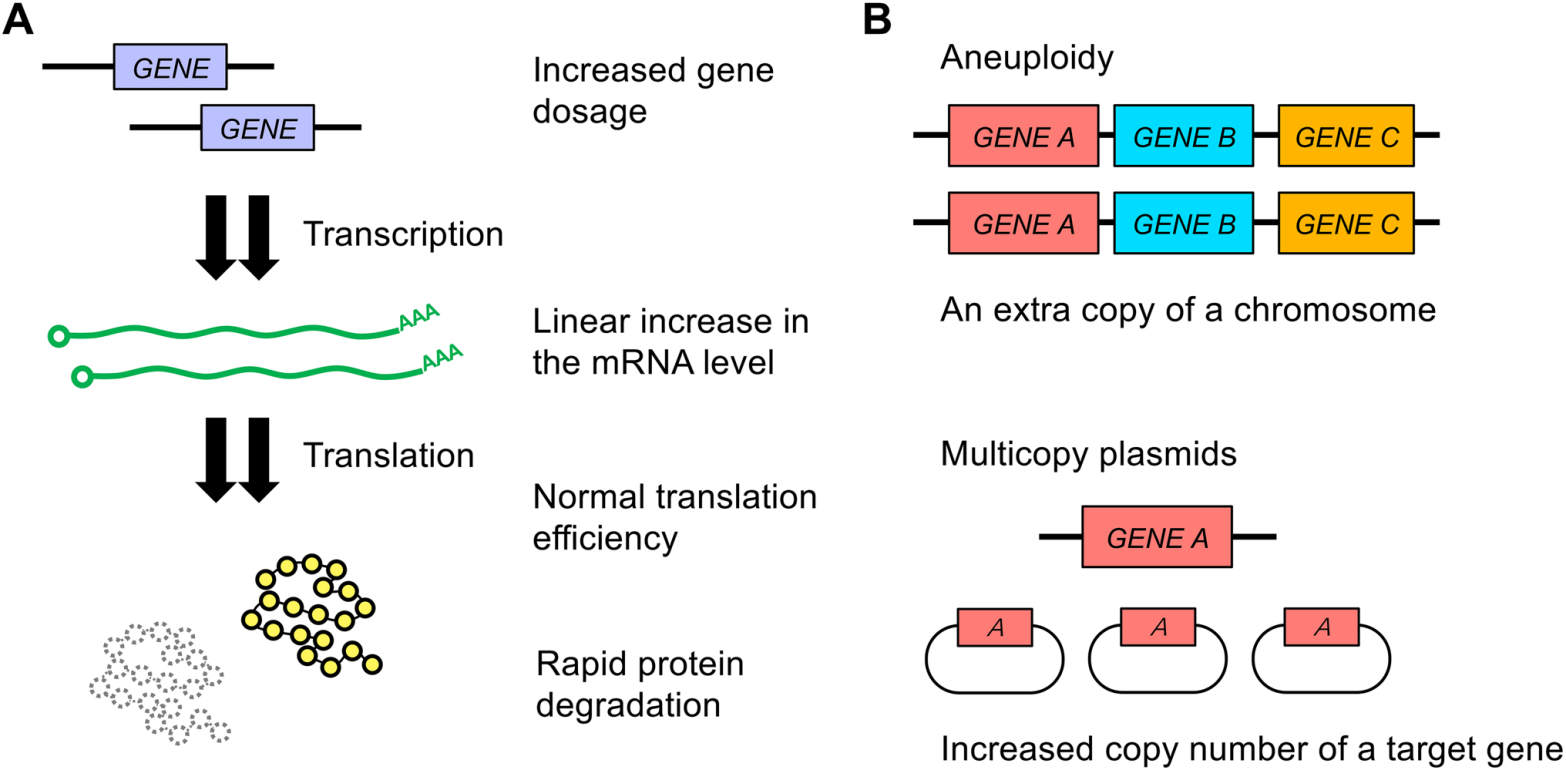
Protein-level dosage compensation. **A** Protein-level dosage compensation buffers genetic perturbations post-translationally. **B** Genetic perturbations caused by increased gene dosage due to aneuploidy and multicopy plasmids. They affect cellular systems differently (Bonney et al. 2015), and not all of the compensated proteins identified in these conditions overlap (Ishikawa et al. 2017)

An increase in gene dosage does not necessarily lead to a linear increase in protein level, which is mediated by protein-level dosage compensation that buffers gene dosage effects at the protein level rather than the mRNA level (Fig. 1A). For example, in *S. cerevisiae*, (i) changes in gene expression by a transient imbalance in gene copy number during meiosis are buffered post-translationally, which is mainly observed for genes encoding multiprotein complex subunits (Eisenberg et al. 2018); (ii) in aneuploid cells (Fig. 1B), the increase in protein levels of genes on an extra chromosome is not necessarily twofold, and these proteins are also enriched in subunits of multiprotein complexes (Dephoure et al. 2014; Torres et al. 2007); (iii) the complex subunits encoded by paralogous genes are likely compensated for modulating protein interactome (Diss et al. 2013, 2017); (iv) genetic screening using multicopy plasmids also revealed that 5 out of 54 tested genes on chromosome I are subject to dosage compensation (Fig. 1A and B) (Ishikawa et al. 2017). These results indicate that protein-level dosage compensation plays a role in buffering gene dosage effects under unperturbed and perturbed conditions.

Systematic identification of the compensated proteins revealed that around 10–20% and 25% of the genome and 70% and 57% of genes encoding subunits are subject to dosage compensation in yeast and human cells, respectively (Dephoure et al. 2014; Ishikawa et al. 2017; Stingele et al. 2012). This finding is consistent with the ‘balance hypothesis’ that links stoichiometric imbalance of the complex subunits and a negative impact on cell growth (Papp et al. 2003; Veitia et al. 2008). Indeed, overexpression experiments have confirmed this hypothesis in 13 out of 49 tested subunit pairs (27%) (Makanae et al. 2013). It is unclear whether the other 36 complexes are compensated, thereby avoiding growth defects, but recent results suggest that dosage compensation contributes to cellular robustness (Ascencio et al. 2021). The effect of gene duplication on fitness was measured for 899 essential genes in budding yeast, and it was found that only about 10% of the genes were associated with fitness disadvantage and that the effect on fitness was limited because genes encoding subunits were protected by dosage compensation (Ascencio et al. 2021). Therefore, it is concluded that the primary function of protein-level dosage compensation is to fine-tune stoichiometry of multiprotein complex subunits.

Since not all subunits are subject to dosage compensation, there may be conditions that determine their potential to be substrates. The assembly interface of the unassembled subunit is free from the partner subunit, and the number of direct interactions with partner subunits should reflect how much of the surface area of unassembled subunit is occupied by non-interacting parts. Theoretically, subunits with a higher number of interactions are more unstable when unassembled than subunits with fewer interactions (Veitia et al. 2008). Thus, the number of interactions seems to be one factor in determining the substrate of dosage compensation, which is supported experimentally (Ishikawa et al. 2017; Mueller et al. 2015).

## Mechanisms of protein-level dosage compensation

Ribosome profiling experiments have shown that there is a high correlation between translation efficiency of mRNAs encoding complex subunits and their stoichiometry (Li et al. 2014). This phenomenon ‘proportional synthesis strategy’ has been observed in bacteria, yeast, zebrafish, mouse, and human cells, indicating that regulation of protein synthesis generally contributes to stoichiometry control under physiological conditions (Taggart and Li 2018). However, proportional synthesis is not always sufficient for proper stoichiometry because translation efficiency is matched within roughly 20% and this difference is considerably large, especially for highly expressed proteins (Li et al. 2014). For example, in yeast data set containing well-characterized complexes, two out of six subunits of the signal recognition particle are synthesized in excess. Intriguingly, ribosome profiling has revealed that translation efficiency of mRNAs encoding subunits of multiprotein complexes does not decrease with increasing gene dosage, excess proteins are synthesized with normal translation efficiency, and they are rapidly degraded by the ubiquitin–proteasome system (Fig. 1A) (Dephoure et al. 2014; Eisenberg et al. 2018; Ishikawa et al. 2017; Taggart and Li 2018; Thorburn et al. 2013).

**Fig. 2:**
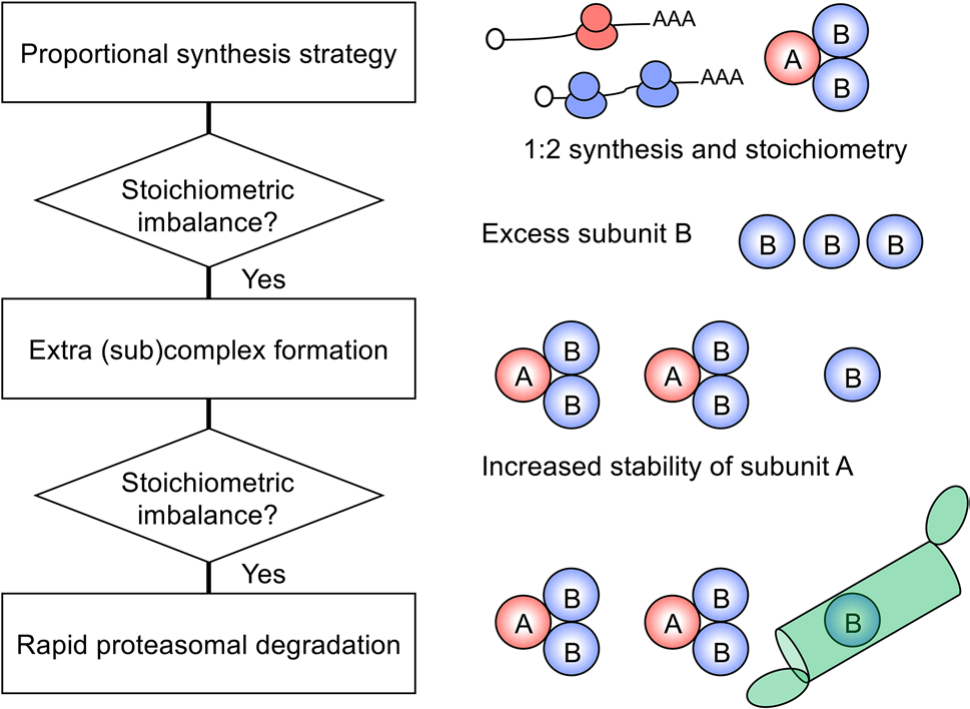
Multilayered structure of the stoichiometry control system. The first layer is the proportional synthesis strategy that enables scaling between stoichiometry and translation efficiency. An exemplary protein complex with 1:2 stoichiometry is shown. If translation efficiency is not proportional to stoichiometry of the complex, or if excess subunits are synthesized due to increased gene dosage, the next layers correct stoichiometric imbalance. The second layer is to control the amount of (sub)complex, thereby stabilizing the partner subunits. The third layer is the rapid degradation of excess subunits by the ubiquitin–proteasome system

The half-life of physiologically over-synthesized subunits tends to be shorter than that of proportionally synthesized subunits (Taggart and Li 2018), consistent with several studies showing that degradation of unassembled subunits by the ubiquitin–proteasome system is the compensation mechanism (Figs. 1A and 2). First, the compensation is generally reduced in the presence of the proteasome inhibitor MG132 (Dephoure et al. 2014). Second, polyubiquitinated forms of the compensated proteins accumulate in the proteasome-deficient mutant (Ishikawa et al. 2017). Third, yeast E3 ubiquitin ligases Doa10, Not4, and Tom1 and a mammalian E2 ubiquitin-conjugating/E3 ligase hybrid protein UBE2O were identified to be responsible for stoichiometry control (Hwang et al. 2010; Shemorry et al. 2013; Sung et al. 2016; Yanagitani et al. 2017). Multiple E3 ligases (Not4 and Tom1) are rarely involved in the compensation of the same subunits (Ishikawa et al. 2020). Identifying such combinations and other compensation E3 ligases is particularly promising for a better understanding of the compensation mechanisms. Likewise, because nuclear-localized E3 ligase Tom1 tends to compensate nuclear proteins (e.g., histone H2A/H3/H4, ribosomal proteins, and the RNase P/MRP subunits) (Ishikawa et al. 2020; Singh et al. 2009; Sung et al. 2016), it is also important to determine the substrate preference of the compensation E3 ligases.

Aneuploid cells are prone to form protein aggregates (Oromendia et al. 2012), which is mainly due to stoichiometric imbalance of protein complexes (Brennan et al. 2019). This study has also revealed that both aggregate formation and proteasomal degradation are involved in the mechanism of stoichiometry control and that dosage compensation of the same protein by both pathways rarely occurs (Brennan et al. 2019). Aggregation of excess subunits may protect cells from gene dosage effects that could cause inappropriate protein-protein interactions (Levy et al. 2012). However, the fate of aggregated excess subunits is unclear, and it remains to be investigated whether they are eventually degraded. The degradation of such large aggregates is unlikely to be mediated by the ubiquitin–proteasome pathway; rather, selective autophagy seems to play more important roles in aggregate clearance (Tyedmers et al. 2010). Although p62-mediated autophagy seems to be active in aneuploid human cells (Stingele et al. 2012, 2013), there is no direct evidence for selective degradation of unassembled subunits through this pathway. Because mouse but not yeast aneuploid cells are sensitive to the autophagy inhibitor chloroquine (Tang et al. 2011; Torres et al. 2007), it might be possible that autophagy-mediated dosage compensation occurs specifically in aneuploid mammalian cells.

Another point to consider is the fact that the stability of a subunit protein is affected by its partner subunit; the amount of the Rbg1–Tma46 heterodimer changes in response to the amount of *TMA46* (Ishikawa et al. 2017). This regulation may occur also at the subcomplex level, as (i) the analysis of the Pop6–Pop7 subcomplex of the RNase P/MRP complexes has shown that overexpression of Pop6 stabilizes Pop7 and vice versa (Ishikawa et al. 2020), and (ii) deletion of the gene encoding oligosaccharyltransferase (OST) complex subunit destabilizes other subunits of the same OST subcomplex (Mueller et al. 2015). These results suggest that stoichiometry is controlled at the subcomplex level before multiple subcomplexes are assembled into a fully formed multiprotein complex, providing insight into the layered structure of the stoichiometry control system (Fig. 2). Whether these extra subcomplexes will eventually be degraded or assembled into functional complexes remains to be investigated, but the stoichiometric imbalance could be fine-tuned by changing the amount of subcomplexes as well as their degradation by the proteasome. Indeed, degradation rates of subunits belonging to the same subcomplex of the OST complex influence each other (Mueller et al. 2015). This observation has suggested that stoichiometry of this complex is fine-tuned by both the second and third layers (Fig. 2) and that the stability of the subunit corresponds to the state of assembly: (stable) fully assembled complex ≥ subcomplex > unassembled monomeric subunit (unstable). It is further supported by the observation of a similar degree of dosage compensation between subunits of the same subcomplex of the 26S proteasome (Ascencio et al. 2021). Notably, the second layer does not apply to dosage-sensitive complexes including tubulin, whose excess is associated with toxicity, and thus its stoichiometry is tightly controlled in the first layer (Katz et al. 1990; Li et al. 2014; Makanae et al. 2013).

## Recognition of unassembled subunits for selective degradation and assembly control

**Fig. 3:**
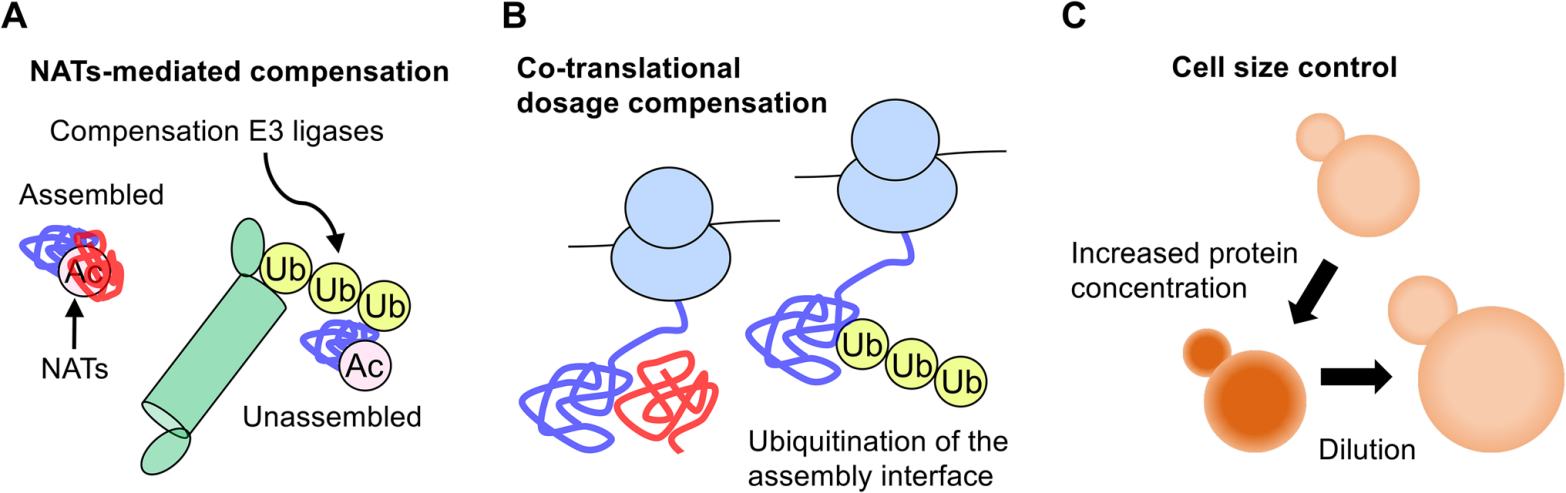
Possible mechanisms that regulate proteome stoichiometry and intracellular protein concentration. **A** Selective degradation of unassembled subunits by the Ac/N-end rule pathway. The N-degron of assembled subunits is shielded by partner subunits of the same complex and is not targeted by N-recognin, while that of unassembled subunits is accessible and enables selective degradation. **B** A ‘lysine hide-and-seek’ model for co-translational dosage compensation that degrades nascent polypeptides emerging from ribosomes translating mRNAs encoding excess subunits. This model is based on the assumption that in the absence of a co-translationally assembled partner subunit, lysine residues at the assembly interface of nascent polypeptides are ubiquitinated and subsequently degraded by the proteasome. **C** A model for regulating intracellular protein concentration by controlling cell size. When the increase in protein concentration exceeds the degradation capacity (e.g., proteasome overload), protein concentration is diluted and proteome stoichiometry is maintained constant by increasing the cell size

While evidence for degradation-mediated stoichiometry control is accumulating as described above, how cells recognize and selectively degrade unassembled subunits is not well understood. The Ac/N-end rule pathway is one proposed mechanism by which N-terminal acetylation of proteins acts as a degradation signal (N-degron) that could be recognized by the compensation E3 ligases, Not4 and Doa10 (N-recognin) (Fig. 3A) (Hwang et al. 2010; Shemorry et al. 2013; Varshavsky 2011). Dosage compensation by this pathway is based on the assumption that N-degron of assembled subunits is shielded and inaccessible by N-recognin when in complex with the other subunits in the same complex, while that of unassembled subunits is exposed and accessible by N-recognin.

N-terminal acetylation is a widespread protein modification mediated by N-acetyltransferases (NATs), accounting for about 68% and 85% of the yeast and human proteomes, respectively (Van Damme et al. 2011). In yeast cells, there are five NATs (NatA–NatE) bound to the ribosome, and in principle each NAT acetylates specific N-terminal residues, which has been reviewed extensively (Aksnes et al. 2016, 2019; Starheim et al. 2012). N-acetylation is found in the majority of proteomes, but the extent to which NATs-mediated compensation is widespread and contributes to proper stoichiometry has not been investigated. Recently, we examined whether the compensated proteins are stabilized in the absence of the catalytic subunit (Naa10–Naa50) of each NAT and found that the contribution of NATs to the compensation varies among subunits (90% for 2 and 10–40% for 12 out of 14 subunits) (Ishikawa et al. 2020). These two proteins, Pop3 and Bet4, were almost fully uncompensated in *naa40*∆ cells, although only histone H2A/H4 are known as substrates of N-acetylation by Naa40 (Song et al. 2003). These histone subunits were not stabilized in *naa40*∆ but less compensated in *tom1*∆ cells, suggesting NATs-independent stoichiometry control (Ishikawa et al. 2020). In contrast, Rmp1 and Bet4 were compensated via multiple NATs; Rmp1 was compensated to a lesser extent in the absence of Naa20, which is responsible for its N-acetylation, compared to Naa10. A comprehensive analysis of dosage compensation using mutants lacking multiple NATs is needed to investigate their functional interactions and working principles in the stoichiometry control system. We also found that NATs-mediated compensation becomes relevant when its target protein is overexpressed, consistent with the observation that N-acetylation rarely acts as a degron under physiological conditions (Kats et al. 2018). These results suggest that the Ac/N-end rule pathway is partially responsible for stoichiometry control.

Several different strategies exist for controlling the assembly of proteins localized to the endoplasmic reticulum (ER). The unassembled subunit of the Na/K-ATPase in *Xenopus* oocyte is retained in the ER and associates with the ER chaperone BiP until it is complexed with partner subunits (Beggah et al. 1996). The binding of BiP reduces the degradation of unassembled subunits of this complex and increases its stability. Another strategy is to control the assembly interface between subunits. Recently, a ubiquitously expressed kinase WNK1 has been identified as an assembly factor for the ER membrane complex (EMC) in human cells (Pleiner et al. 2021). This study has revealed that WNK1 selectively binds to unassembled EMC2 and competes with the binding of E3 ubiquitin ligases (MKRN1 and HUWE1) to protect it from degradation. WNK1 seems to dissociate from EMC2 when the partner subunit is available for assembly (Pleiner et al. 2021), which may be another strategy in the second layer of the stoichiometry control system (Fig. 2). Thus, identifying factors responsible for this type of assembly control will also facilitate an understanding of how cells regulate proteome stoichiometry.

## Perspectives

An important issue arising from previous studies is quality control of excess subunits at the co-translational level. One plausible model is that failures in complex assembly are monitored co-translationally by assembly factors that bind to nascent polypeptides emerging from the ribosome, as proposed in (Juszkiewicz and Hegde 2018). On the other hand, co-translational degradation of nascent polypeptides with N-degron in the Arg/N-end rule pathway (Turner and Varshavsky 2000) raises another possibility that protein-level dosage compensation occurs even during the synthesis of unassembled subunits. Co-translationally ubiquitinated nascent polypeptides undergo proteasomal degradation, which is present in yeast and mammalian cells, but its functions remain unclear (Duttler et al. 2013; Wang et al. 2013). Given that cells can recognize whether the assembly interface of nascent subunit emerging from the ribosome is shielded by a partner subunit, one possible mechanism for co-translational dosage compensation might be ubiquitination of lysine residues at the unpartnered assembly interface of excess subunits (Fig. 3B). Controlling the complex stoichiometry by this mechanism would be cost-beneficial because the absence of one subunit leads to aggregation of a co-translationally assembled partner subunit (Shiber et al. 2018). The compensation E3 ligase Not4 binds to translating ribosomes (Dimitrova et al. 2009; Halter et al. 2014; Preissler et al. 2015); however, deletion of Not4 doubles the amount of co-translationally ubiquitinated nascent polypeptides, suggesting that Not4 may not contribute to co-translational ubiquitination (Duttler et al. 2013). Co-translational ubiquitination is partially reduced in the absence of Hul5, Hrd1, or both Hel2 and Ltn1 (up to 25% in *hel2*∆ *ltn1*∆ cells) (Duttler et al. 2013). This reduction in the double mutant seems to be due to the lack of ribosome-associated quality control, which is required for degradation of aberrant nascent polypeptides by the ubiquitin–proteasome system (Brandman and Hegde 2016; Joazeiro 2019), but the functional impact of the other fraction of co-translational ubiquitination remains an important question.

While many studies have shown that dosage compensation mainly targets subunits of multiprotein complexes as described above, evidence is accumulating for the global control of intracellular protein concentration by the regulation of cell size (Fig. 3C). Examples include: (i) overexpression of a positive regulator of mammalian cell size, called Largen, promotes translation of mRNAs, including those encoding histone and mitochondrial proteins (Yamamoto et al. 2014); (ii) overexpression of unneeded exogenous protein (mCherry) results in an increase in cell volume as well as endogenous protein levels in *S*. *cerevisiae* (Kafri et al. 2016); (iii) aneuploidy leads to a larger cell volume, which seems to be correlated with increased protein expression due to an extra chromosome (Thorburn et al. 2013); and (iv) cyanobacteria maintain constant protein concentration when genome copy number is increased, which coincides with an increase in cell volume (Zheng and O'Shea 2017). Because proteotoxicity is thought to result from an overload of degradation machinery (Harper and Bennett 2016; Moriya 2015; Oromendia and Amon 2014), perhaps it is more advantageous to regulate cell size than to enhance degradation to survive under such challenging conditions. Therefore, a quantitative study of the relationship between mRNA translation and protein degradation would provide insight into the mechanism by which protein concentration is optimized by regulating cell size.
